# 16,648 reasons to live instead of dying by suicide: insights from a computer-assisted content analysis

**DOI:** 10.1038/s41598-026-58537-4

**Published:** 2026-07-07

**Authors:** Philip Resnik, Rebecca Resnik, Katherine M. Schafer, Josh Hagedorn, Jonathan B. Singer

**Affiliations:** 1 Department of Linguistics and Institute for Advanced Computer Studies, University of Maryland, College Park, MD 20814 USA; 2 Rebecca Resnik and Associates, North Bethesda, MD 20852 USA; 3 Vanderbilt University Medical Center, Nashville, TN 37232 USA; 4 TBD Solutions, Grand Rapids, MI 49546 USA; 5 School of Social Work, Loyola University Chicago, Chicago, IL 60660 USA

**Keywords:** Suicide prevention, Lived experiences, Qualitative content analysis, Computer assisted methods, Health care, Psychology, Psychology

## Abstract

Most research on suicide focuses on the progression toward lethal action. Fewer studies have looked at individuals’ past experiences with the desire to die and why they did *not* die by suicide. Moreover, the existing use of reasons to live in assessment and treatment is generally grounded in inventories of questions that, while groundbreaking and well validated, were developed decades ago and without a focus on individuals’ lived experiences. In this study, an online user’s query to formerly suicidal people on the popular Reddit platform afforded a novel opportunity to investigate reasons people lived in a large, naturally occurring sample of 16,648 self-reports about their experiences. Using a new method for computer-assisted qualitative content analysis, we identify categories, and themes organizing those categories, that affirm prior work and also provide new perspectives on that work, as well as suggesting connections between ideas in the literatures on reasons people die, reasons people live, and subjective and psychological well-being. The study highlights the value of computer-assisted methods as a way of achieving both scale and interpretable results, and it identifies a number of theoretical and clinical avenues for further investigation.

On September 27, 2020, a user called firegate2233 posted on AskReddit, a discussion forum on the popular Reddit site. He asked, “Formerly suicidal redditors, what’s something that kept you alive a little while longer and helped you to get through the dark times in your lives?”. Within 24 hours, the question had amassed more than 15,000 responses.

When it comes to trying to understand suicidal thoughts and behaviors, the great majority of research is concerned with identifying and assessing factors that bring people *to* suicidal ideation, attempts, and death. (Use of the term *suicidality* varies in the literature. Here and throughout, our use of the term and its derivatives is intended to encompass suicidal thoughts, ideation, plans, suicide attempts, and death by suicide^1^). Dominant theories^2–8^ cluster together in a framework called “ideation to action”, which focuses on the progression from suicidal thoughts to life-threatening suicide attempts^9^, and proposals for near term assessment of suicidal crisis also focus on considerations associated with dying^10,11^, as do technological approaches for risk assessment^12–15^. Far fewer studies have looked at reasons people do *not* die by suicide, and there work has tended to be less theoretical and more treatment-centered. In particular, the widely used Reasons for Living inventory^16^ RFL,] and elicitations of people’s reasons to live, play an important role in frameworks for suicide risk management and safety planning^17–20^. Since the publication of the RFL scale, however, most scholarship on reasons for living has been conducted in clinical settings^21^, and most studies involve small samples^16,22–28^.

In this context, what makes firegate2233’s question particularly interesting is not only that it is focusing on why people *didn’t* die by suicide, but that it yielded a *large*, *diverse*, and *recent* dataset of responses with the potential to yield novel insights into how people manage to put the brakes on the progression from ideation to action. The Reasons for Living inventory is well established, well validated, and still central in active research^29^, but it was constructed 40 years ago in a very different sociocultural context and it asked individuals to *list reasons* for not dying by suicide. Firegate2233’s phrasing and the context of AskReddit encouraged more narrative replies, raising the possibility that new insights might emerge in a more current and experience-focused examination of the question. In our dataset the mean response length is a paragraph-like 62 words, versus RFL items’ bullet-item-like 10 words^16.^

More generally, the responses to firegate2233’s question about “what kept you alive a little longer” pertain to a less-studied aspect of the suicidal experience. Figure 1 organizes suicide research into four quadrants on two dimensions: reasons people die versus reasons people live, and longer-term versus near-term risk. The first dimension captures the widespread framing of suicide risk in terms of opposing forces pushing toward death and life^5,9,30,31^. The second dimension distinguishes research on the considerations characterizing suicide risk over longer time periods^32^ from those associated with acute suicidal crisis and short time scales^10^. These distinctions are not strict—for example, the Narrative Crisis Model^33^, fluid vulnerability theory^34^, and ideation-to-action theories^9^ engage with transitions from longer- to near-term risk, and the CAMS treatment framework^18^ explicitly elicits both reasons for dying and reasons for living. However, this framing of the research highlights the contrast between decades of study in the top quadrants versus relatively newer approaches in the bottom quadrants, as well as the contrast in research volume for risk factors on the left as opposed to protective factors on the right. Our dataset provides a unique opportunity to gain insights within the quadrant that has been least studied.

**Fig. 1:**
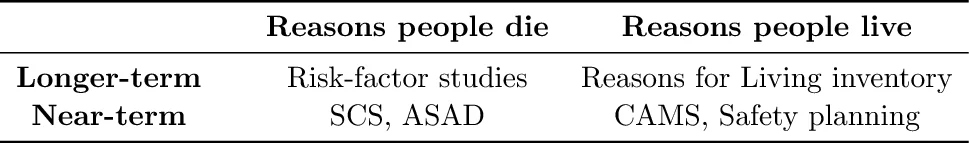
Four-quadrant framework for understanding suicide, with (non-exhaustive) examples.

The present study used a computer assisted form of inductive content analysis^35^ to develop categories and themes from the responses firegate2233 received. As we discuss below, the technique uses a computational model called Latent Dirichlet Allocation^36^ to automatically discover “topics”, each of which is characterized by probability distributions over words and items of text; those topics then drive an efficient, carefully structured qualitative analysis by subject matter experts. Thanks to the hybrid human-computer approach, we were able to include a full 16,648 text responses in our analysis in a process that took only hours from beginning to end. This contrasts with the sampling-down by several orders of magnitude that has previously been required for manual content analysis of similar data^37^.

## Materials and methods

### Dataset

We collected responses to firegate2233’s question via automated downloads using the PushShift API, filtering to include only top-level responses to the original question because secondary responses (i.e. comments on others’ responses) did not reliably reference reasons for living, often providing commentary or emotional support to top-level responses. The dataset was compiled on October 28, 2020 and includes posts made over a period of 26 days (from September 27 to October 10, 2020). The final, filtered dataset of top-level posts included 15,314 distinct entries, of which 15,073 (98.4%) were made within 24 hours of firegate2233’s initial query.

In addition to the firegate2233 data, we explored whether there might be previous AskReddit threads with similar content, and identified three that were substantially similar, albeit with substantially fewer responses—1,334 total. Given the qualitatively similar nature of the questions and responses, we chose to include the additional data to produce a final dataset containing 16,648 posts in which previously suicidal Reddit users responded directly to a question asking what had prevented them from dying by suicide. (We conducted the automatic part of our analysis using both the full set of 16,648 posts and the 15,314 responses to firegate2233, and the results were extremely similar. Our results in the remainder of the paper are based on the full 16,648-item dataset.) Posts vary widely in length, from a single word to 1362 words. The average length of posts in the dataset is 62.3 words, with a standard deviation of 85.3.

Because participation was self-selected and not restricted to any defined population, our dataset comprises a nonprobability convenience sample of Reddit users. This limits claims to generalizability, although, as we note in discussing the results, our analysis of the data provides evidence of convergent validity and therefore a solid grounding for future work using systematically obtained samples. About 24% of U.S. adults report using Reddit, highest among adults aged 18–29 (used by 46%) and 30–49 (used by 35%); men are slightly more likely than women to use Reddit (used by 28% vs. 20%), and usage rises with education and income, reaching 32% among college graduates and 34% among adults earning over $100,000 annually^38^.

### Analysis method

We conducted a bottom-up or inductive form of qualitative content analysis^39^, an approach commonly used in the social sciences. In light of the practical challenges of traditional inductive content analysis methods—which would already be labor intensive with a set of a thousand short responses, much less sixteen thousand longer ones—we employed a novel computer assisted method that we recently developed for analysis of open-ended responses in survey research TOPCAT, Topic Oriented Protocol for Content Analysis of Text;^35,40^, which is grounded in topic modeling^41^.

Topic models are a class of automatic, unsupervised methods for discovering categories in collections of text, and therefore they can serve a purpose similar to the manual process of traditional, inductive category discovery in qualitative content analysis^42–45.^ (In machine learning, the term *unsupervised* refers to methods that do not require examples of correct analyses, e.g. pre-coded items, in order to learn useful things about the data.) As a source of high level intuition, topic modeling can be viewed as similar in spirit to principal component analysis (PCA), but using unstructured text documents (e.g. Reddit posts) as the items, and using the words in those documents (more generally a vocabulary of words and phrases) as the measurable features of those items^46^. The most widely used form of topic modeling, Latent Dirichlet Allocation^36^, yields a set of latent categories, each represented as a probability distribution over the vocabulary, and these are analogous to the latent principal components discovered by PCA. LDA also infers a probability distribution over those categories for every document, which is analogous to the way that PCA analysis assigns a “loading” on each component to every item.

**Fig. 2:**
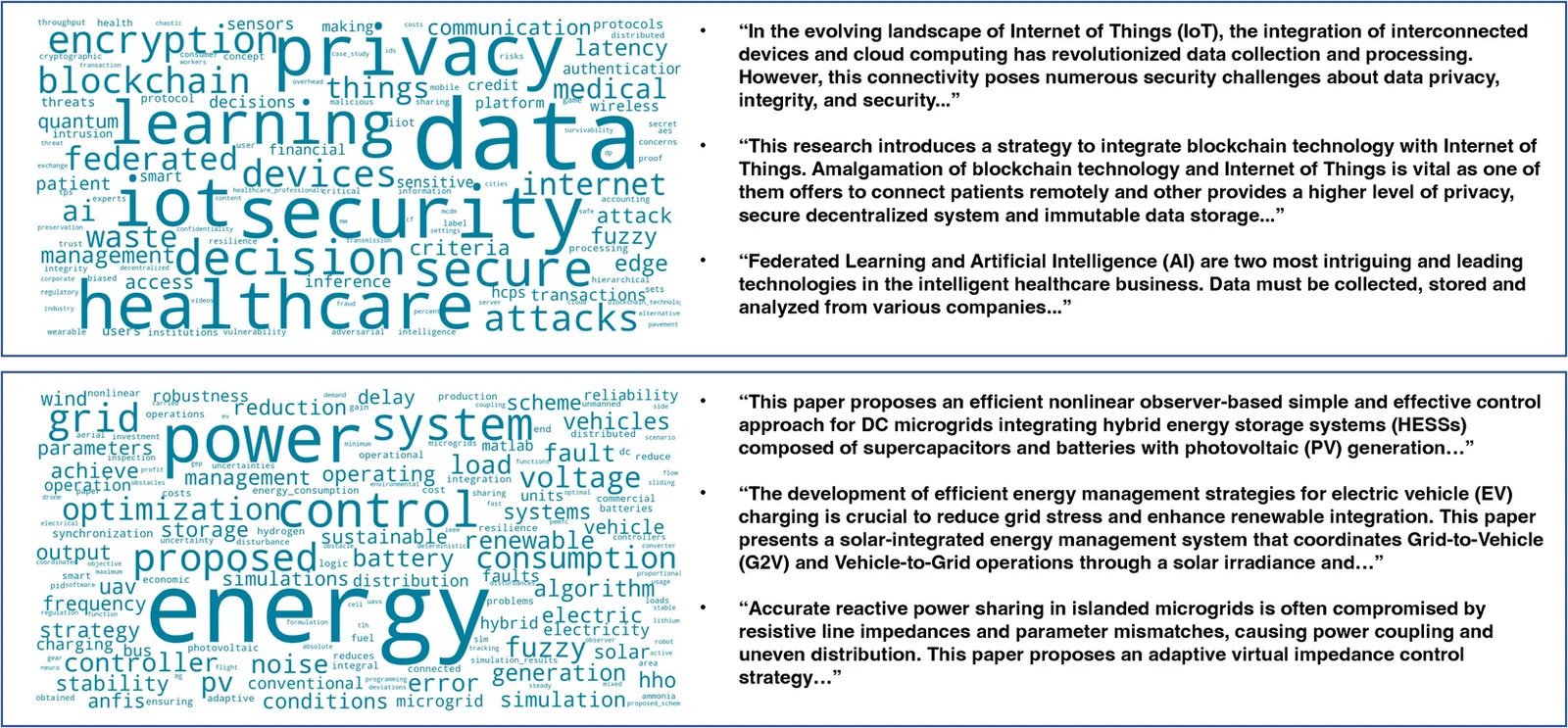
Illustrative visualization of two LDA topics from topic modeling with a sample of *Scientific Reports* abstracts. The top topic surfaces a category one could label *Data Privacy, Integrity, and Access*, where representative documents focus largely on “Internet of Things” (IOT) connected systems, especially in healthcare applications. The bottom topic captures a category one might label *Power Systems Control and Renewable Energy Optimization*, which covers optimization and control strategies for power systems that integrate renewable energy sources, storage (batteries, PV, fuel cells), and grid-connected infrastructure.

These sets of probability distributions are what make LDA so powerful as a tool for human interpretation of underlying category structure in text collections: if a latent topic gives highest probability to words like *god*, *faith*, *religious*, *hell*, *jesus*, *christian*, for example, and documents loading most strongly on that topic include statements like “I come from a Christian community where people have been taught to fear hell and eternal damnation”, then it is straightforward for a human content analyst to infer that this is a category related to religion. Representing LDA topics in a convenient visual form for content analysts is straightforward: a topic *t*’s probability distribution over words, *\Pr (W|t)*, translates very naturally into a word cloud or bar chart visualization, and ranking the set of documents by *\Pr (t|d)* provides a natural way to identify highly ranked documents *d* that can be thought of as representative of the topic. Figure 2 illustrates with two topics from an LDA model that was created using a sample of *Scientific Reports* abstracts from December 2025.

Topic modeling has a number of advantages over traditional qualitative content analysis. It proceeds bottom-up from expressed language, like the traditional inductive process in QCA. However, being fully automatic, it avoids biases that human content analysts might bring to the data. For similar reasons it is more replicable than manual analysis, and it is vastly more scalable, permitting efficient analysis of thousands or even hundreds of thousands of documents. In addition, a stable and mature implementation of LDA is widely available in the widely used MALLET toolkit^47^ and there are excellent resources supporting its use suitable for non-specialists [e.g.,]^48^, enhancing the replicability of our approach and encouraging its use by others.

Like any other fully automatic technique, however, topic modeling can produce a mix of good and bad results—in particular, not all topics are relevant and interpretable. Our TOPCAT protocol is an intuitive, efficient way to bring humans into the loop in order to add a layer of quality control and interpretation to the topic modeling process.

TOPCAT can be viewed as a highly scalable form of inductive thematic analysis^39^. It has five steps, once the collection of texts to analyze is in hand. (The full TOPCAT protocol, including supporting software and instructions for the human curation process, is publicly available on GitHub at https://github.com/psresnik/topcat/).

- Preprocessing. The text is preprocessed automatically using natural language processing techniques, e.g. to identify multi-word phrases such as *video_game* or *opioid_addict*, and to normalize text in standard ways like removing punctuation and “stoplist” words (very common words like *the*, *another*, etc.).
- Granularity selection. A set of several topic models is built automatically at multiple granularities, ranging from coarser- to finer-grained. One model is then selected as the starting point for human curation.
- Independent curation. Two subject matter experts independently follow a set of detailed instructions for reviewing and curating the proposed categories, resulting in two sets of independently determined category labels/descriptions. This is analogous to independent creation of two distinct codebooks for the same data, driven by the topics proposed by the model. Crucially, this step includes a strict limit on the time spent per topic, which is possible because model visualization guides analyst attention to the most salient aspects of each model-proposed category.
- Creating consensus categories. The two sets of categories are combined, informed by subject matter expertise and the data, to create a “consensus” set of categories. This step is also time-limited and focuses on the results of curation in the previous step, not further analysis of the original items.
- Grouping into themes. Optionally, the consensus categories are manually curated into higher-level themes.

During the independent curation and consensus categories steps, the TOPCAT protocol permits discarding model-proposed categories that are insufficiently interpretable or irrelevant, and combining multiple model-proposed topics if they describe the same conceptual category.

## Results

Our computer assisted content analysis protocol yielded 23 consensus categories, which we then manually organized into four themes. During that process, we observed that one of the categories, “Hope and connection with and/or fear, from spiritual or religious beliefs”, constituted a combination of two distinct categories with opposite valence, a not-uncommon issue in topic modeling since LDA models capture what texts are *about* rather than associated emotion or sentiment. We split this into two categories, “Hope and connection with spiritual or religious beliefs” and “Fear related to religious beliefs” each of which fits naturally into one of the four thematic groupings. As Yardley and Marks^49^ observe, “no theme can be entirely inductive or data driven, since the researchers’ knowledge and preconceptions will inevitably influence the identification of themes”. Here, we emphasize, that fact is not a bug in the process, but a feature.

### Qualitative analysis: themes and categories

*Theme 1: Concern for others.* *Categories related to taking care of others in a broad sense: respondents decided not to attempt suicide because they did not want to hurt, burden, or cause trauma to their loved ones, or because they wanted to continue taking care of those they loved.*

1. Not wanting to hurt specific family members
2. Financial burdens for survivors or if attempt fails
3. Not wanting to traumatize loved ones, especially finding and having to deal with the body
4. Pets, especially cats and dogs – not wanting to hurt them, worry about them, etc.
5. Not wanting to hurt specific family members, particularly children

**Fig. 3:**
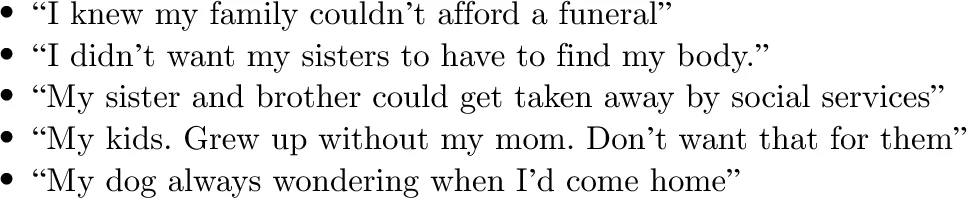
Illustrative responses in the concern for others theme.

This theme involves considerations that make it harder for someone to believe their loved ones would be better off if they died by suicide—as opposed to viewing themselves as a burden on loved ones who would therefore be better off if they killed themselves. (See Fig. 3 for illustrative examples). Looking at the theme from a theoretical perpective, we note that perceived burdensomeness is a central construct in the influential interpersonal theory of suicide (IPTS), with a solid evidence base developed over the past two decades connecting people’s perceptions of themselves as a burden with suicidal thoughts and behaviors^51^. As responses like “My sister and brother could get taken away by social services” demonstrate, this emergent theme is theoretically well aligned with the Perceived Burdensomeness construct, but from the opposite direction: Concern for Others reflects a recognition that the individual’s *death* would create or constitute burdens for those they care about, and it is therefore a reason to live. (Here and throughout, examples of responses are not verbatim, per widely used guidelines for ethical presentation of dtaa in social media and mental health research [50]. Rather, they are generally excerpted with changes of wording, while retaining the core meaning, to reduce susceptibility to online text searches.)

There is also theoretical overlap here with Klonsky and May’s 3-step theory of suicide (3ST)^52^, which like IPTS focuses primarily on the development of suicidal ideation and the progression toward attempts, albeit with a greater emphasis on the “pull toward life”^5^. In 3ST a central theoretical construct is Connectedness, and concern about others presupposes a sense of connection with them. Klonsky and May conceptualize Connectedness as involving not only connection to others but connectedness to life more broadly—consistent with Mason et al.^37^, who conducted a more traditional small-scale content analysis on similar data, our analysis supports distinguishing the two as separate theoretical constructs. Interpersonal connectedness fits here and categories related to connection to life fit within the theme of positive foresight, as we discuss below.

*Theme 2: Sensory pleasures.* *Categories of response that involve subjective and often transitory pleasure or distraction.*

1. Looking forward to next episodes of TV, new games, experiencing new media
2. Music—wanting to continue hearing it, experiencing music as meaningful, specific artists
3. Food and sensory pleasures
4. Substance use and abuse
5. Spending time on alternative media

**Fig. 4:**
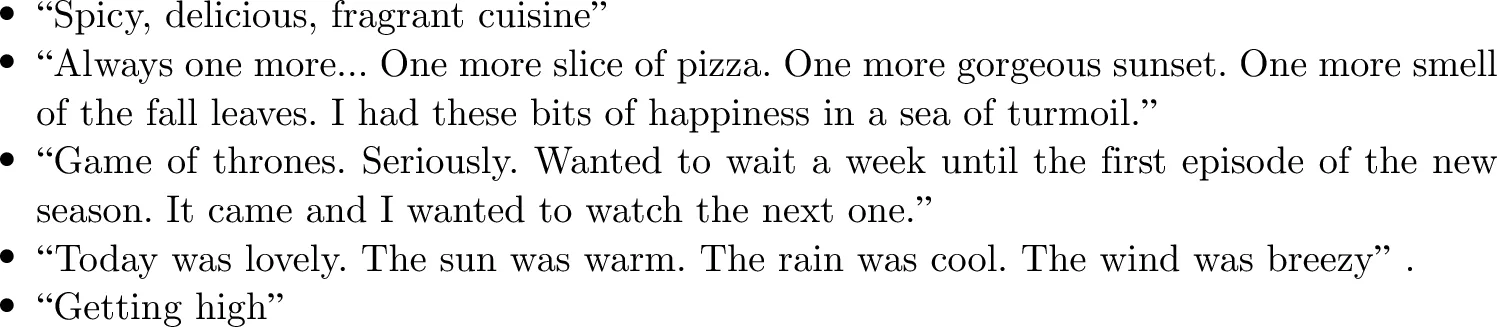
Illustrative responses in the sensory pleasures theme.

This theme connects to the theoretical concept of hedonia, which has typically been discussed in the scholarly literature on well-being and is largely absent from scholarship focused on suicide and its prevention per se. (See Fig. 4 for examples.) Theoretical and clinical research on suicide tends to focus on *an*hedonia— the inability to experience (hedonic) pleasure—and its links to depression and increased risk of suicidal thoughts and behaviors^53–59^. The emergence of this theme in a study of previously suicidal people suggests potential in a unified view bridging the two literatures. (Purists may object to *hedonic* as a modifier of *pleasure*, since the word is the adjectival form of Greek , pleasure, and therefore the phrase would mean *pleasurable pleasure*. However, as we discuss below, hedonia refers to pleasure or happiness in its meaning involving the senses, delight, ecstasy,e.g. sexual pleasure, as opposed to eudaimonia, which involves a “higher sense” that does not refer “simply to a state of present pleasure or enjoyment”^60^. We permit ourselves a bit of redundancy in order to highlight the distinction between the two). 

The evidence here suggests that for some individuals, finding *some* form of pleasurable experience may act as a barrier to the acute anhedonia associated with short-term suicide risk. Hedonic pleasures may also serve as a countervailing force to suicide-specific rumination, which has been identified as being significantly related to suicidal intent^61^ and to loss of cognitive control in suicidal crisis^62,63^. Mak et al.^64^p. 3] note that rumination has “a tendency to shift attention away from the present moment”. Conversely, therapies that reinforce the ability to pay attention *to* the present moment, e.g., rumination-focused cognitive behavioral therapy^65^, have proven effective at reducing rumination over periods of time. Hedonic pleasures may contribute similarly as a distraction that directs the “attentional spotlight” away from repetitive negative thoughts that maintain or exacerbate distress. For example, one respondent wrote,“Music. It’s seems strange but listening to music distracted me from the voices in my head and made me think of other stuff”, another comments that “Books are a great way to escape from reality”, and another, “If I had to feel all my feelings all the time I never would have made it. Gaming gave me an engaging enough distraction to get through each day one at a time”.

*Theme 3: positive foresight.* *In this theme, we find ways that individuals were able to achieve a forward-looking perspective, recognizing the possibility of a meaningful future.*

1. Holding on for one more day and focusing on the next thing, routine, sleep on it and see if/how things get better in the morning
2. Quotes, inspiration, philosophy, wonder—things that resonate/connect
3. Sense of connection—feeling seen, heard, understood, cared about by other people
4. Professional diagnosis and treatment
5. Hope and connection with spiritual or religious beliefs
6. Recognition that suicide is “a permanent solution to a temporary problem” and life will change
7. Hope—looking past the bad and feeling like life can get better
8. Insight/realization about not wanting to die or finding meaning in surviving an attempt
9. Insights from reading or writing, including writing a suicide note
10. Turning point, transition, new connection
11. Finding a new distraction or focus

In general, reasons to live within the theme of Positive Foresight capture ways that individuals managed to get past hopelessness^3,56^ or to go beyond dwelling on negative experiences^6^ to attain a sense of connectedness with life and meaning. This is consistent with suggestions^37,52^ that the construct of Connectedness in theories of suicide should go beyond personal connection^4,6^. Rather than simply broadening the scope of Connectedness to include both, however, we would suggest that Positive Foresight evokes a distinct construct that we associate with *eudaimonic* well-being.

**Fig. 5:**
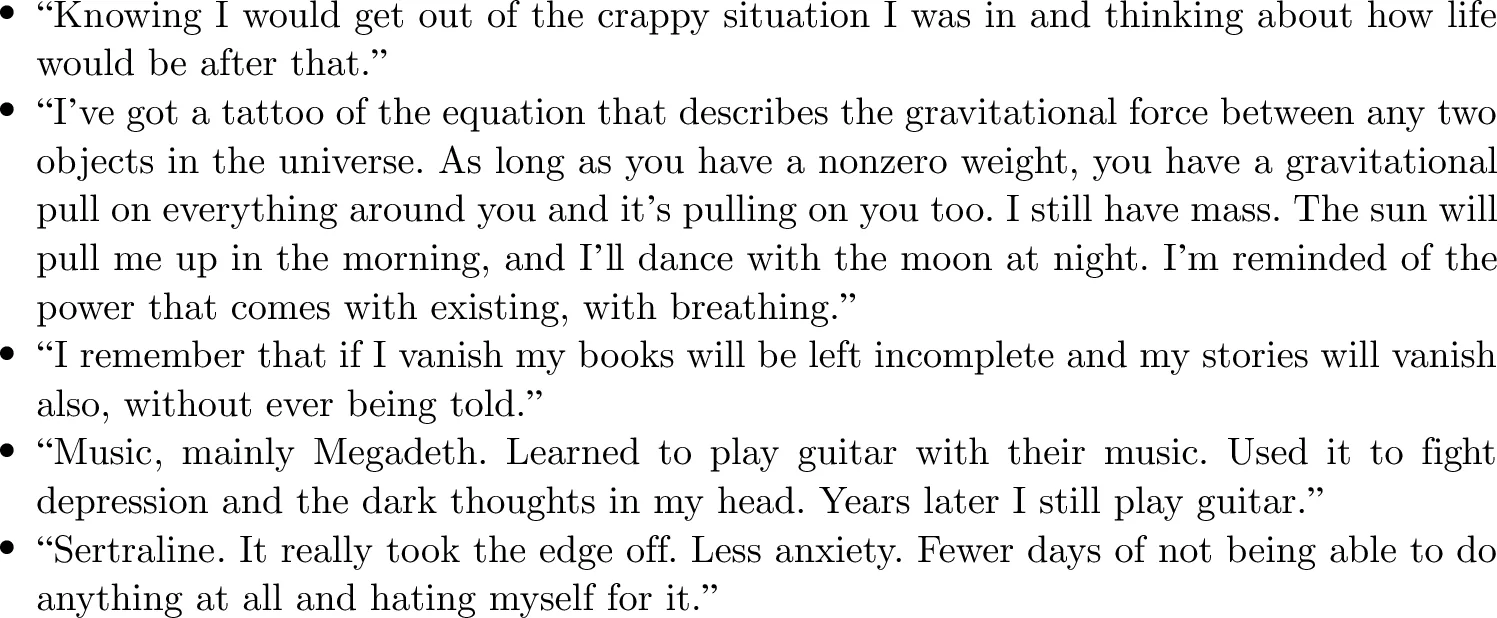
Illustrative responses within the theme of positive foresight.

Eudaimonia is a concept that dates back to Aristotle^66^ and is associated with long-term fulfillment and flourishing; it is often contrasted with hedonia, which typically emphasizes shorter-term pleasure and enjoyment. Like hedonia, eudaimonia tends to appear as an explicit construct in the well-being literature, but it is rare to see it explicitly in scholarship on the progression *toward* suicide. Although eudaimonia still lacks a single widely accepted psychological definition^67,68^, Huta^67^ reviews several lines of modern psychological research and characterizes it in terms of *meaning*, *elevation*, *awe*, *connection*, *aliveness*, *fulfillment*, and *competence*. When these are read in tandem with examples of Positive foresight, strong connections are evident (Fig. 5).

The emergence of this theme suggests that from a theoretical perspective, there is a potentially important and under-studied connection between constructs related to eudaimonia in the well-being literature, on the one hand, and on the other hand constructs in the suicidology literature that are related to generalized connectedness, future orientation, and perception of a meaningful life. (That direction also aligns with studies on “meaning in life” and suicidal behavior, although to date those studies have focused heavily on suicidal ideation^69.^) Just as *anhedonia* is a central symptom of depression and also connected with more immediate suicide risk^56,57,59^, we posit that an analogous construct, which one might term *aneudaimonia*, deserves more attention in the study of suicide.

Note that we do not argue for a *strict* distinction between between sensory pleasures (hedonic) and positive foresight (eudaimonic), since there can be overlaps between the two. For example, consider “Everything about music. Listening to it. Anticipating an upcoming concert. After I joined a band: writing, performing, working to improve my craft.” Here we have the transitory pleasure of enjoying listening to a piece of music, but the pleasure of music also contributes to the author’s having connection to something they are working at for the future. Similarly, one individual credited getting tattoos and piercings as having gotten them through a suicidal period, commenting, “Art on my body made me feel like art, and art deserves to be seen. And you have to take care of tattoos and piercings and that helped get me take care of myself”. This author surely takes pleasure in feeling like a piece of art in transitory moments when they look in the mirror. But at the same time this response evokes the idea of eudaimonic well being in the author’s sense of connection with themselves and the future-oriented, meaningful caretaking of the art on their body. As a third example, consider “Star Trek. A vision of an optimistic galaxy filled with adventure plus sexy starship captains, super short skirts, cool weapons, and a yearning for meaning and knowledge—IN SPACE!”. The author gives reasons they find watching Star Trek pleasurable in a hedonic sense, but also clearly identifies with the way the show is driven by a sense of purpose.

**Fig. 6:**
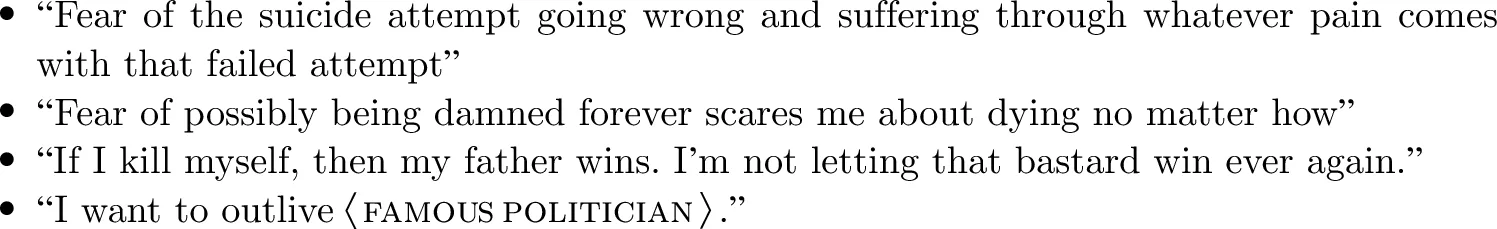
Illustrative responses in the negative valence theme.

*Theme 4: negative valence.* *This theme includes categories connected with negatively valenced emotional or motivational states, such as fear, spite, or anger *(Fig. 6).

1. Fear of surviving an attempt and being worse off
2. Fear related to religious beliefs
3. Spite—proving people wrong

From a theoretical perspective, the emergence in these data of a negative emotion such as spite supports Solomon and Stone’s^70^ critique of a binary or polarity-based framing of emotions as being overly simplistic; in particular, it provides a datapoint in favor of their argument that positive and negative valence should not necessarily be associated respectively with health and illness. It should be emphasized that this does not contradict prior research associating negative emotions with suicide risk. It does, however, suggest a more complex landscape. Within Bryan’s^31^ cusp catastrophe model, for example, a key dimension of the “behavior surface” is characterized in terms of emotional distress, with increases in distress yielding continuous or potentially discontinuous transitions upward on the suicide-risk axis^31^ p. 94]. The spite category, and more generally the negative valence theme, indicate that negatively valenced feelings like spite or anger can also play a *positive* role with regard to suicide risk, e.g. in terms of a focus on the future and motivation to take action. Framed in the context of our earlier discussion, it also suggests that not only positive but some negatively valenced feelings can provide an eudaimonic sense of purpose, meaning, or connection, in contrast to the traditional association of eudaimonia exclusively with virtue or positive characterizations of a meaningful life^66^. Our observation is also consistent with anecdotal report—for example, Linehan^71^p. 45] writes, “When I heard that the hospital was giving up on me *…* I decided that I would prove them all wrong if it was the last thing I did on earth”, continuing that later, “one of my professors told me that this kind of anger could be very helpful in keeping a person from giving up”. To our knowledge analyses of these lived experiences [ours and Mason et al.’s on similar data,^37^] offer the first non-anecdotal evidence for this phenomenon.

### Clinical analysis

Clinical interventions involving reasons to live tend to be prospective: they focus on giving someone who is in treatment the tools going forward to counter the momentum toward choosing to die within “the internal debate of suicide”^23^. In contrast, the individuals whose responses we analyzed may or may not have engaged with the clinical ecosystem at all, and the question they were asked was retrospective: not why they should keep living, but *what got you through* the dark times. We consider here the relationship between our findings and prior clinically-oriented literature, as well as connections and clinical insights our study may offer.

*Breaking the ruminative cycle.* Experiences within the theme of Sensory Pleasures may serve to distract from or redirect the perseverative, ruminative thinking^61,62^ and loss of cognitive control^62,63^ that are often characteristic of suicidal crisis. Mak et al.^64^p. 3] note that rumination has “a tendency to shift attention away from the present moment”, and therapies that counter this tendency, e.g., rumination-focused cognitive behavioral therapy^65^, have proven effective at reducing rumination over periods of time. The evidence here suggests the possibility that hedonic pleasures may contribute similarly to drawing the “attentional spotlight” away from repetitive negative thoughts that maintain or exacerbate distress. For example, one respondent wrote,“Music. It’s seems strange but listening to music distracted me from the voices in my head and made me think of other stuff”; another comments that “Books are a great way to escape from reality”; and another, “If I had to feel all my feelings all the time I never would have made it. Gaming gave me an engaging enough distraction to get through each day one at a time”.

More generally, rumination is associated with exacerbating and sustaining negatively valenced emotional states, increasing negative thoughts, intensifying negative emotional reactions to stressful events, and interfering with problem-solving and attention to current situational context^72^. The contrast between sensory or hedonic pleasures and harmful rumination is consistent with relevant neurobiological distinctions: the brain’s default mode network is connected with introspection and inward focus on thoughts and feelings, and evidence suggests that it operates in a complementary way to the central executive system, which is active when individuals are in a more externally focused, engaged, or problem-solving mode^73^. A shift from default mode to central executive mode activation by focusing on hedonic distractions in the moment may contribute to the underlying cognitive shift that Bryan^31^ describes as “braking”. (Similar shifts at the metacognitive level are a fundamental aspect of cognitive behavioral therapy^74^, and can involve other themes, especially reasons to live with strong emotional salience,e.g. elements of Caring for Others, such as a beloved pet, or of Positive Foresight, such as a compelling professional goal.) With hedonic distraction, an individual may find it easier to shift from interoception to exteroception, from being trapped in self-directed thoughts and feelings to greater autonoetic awareness, facilitating their ability to envision themselves in future that has salience and even meaning. In a sense this insight, about the short-term value of leaning into a transient hedonic experience, can be thought of as complementary to the longer-term therapeutic goal of letting-go of experiences that *should* be transient.

It is worth observing that one of the advantages of our anonymous, naturally occurring, retrospective dataset is that it provides a range of hedonic responses that individuals seem unlikely to share with clinicians. For example, one might be unlikely to list activities that Redditors reported such as masturbation, looking at girls with curly hair, or enjoying hentai, when asked for “things you like doing by yourself” in safety planning^75^, nor when considering reasons for living in contrast to reasons for dying in the CAMS.

*The power of negative thinking.* One of the more surprising themes to emerge in our analysis was negative valence, a category comprising feelings like spite, anger, and fear that has to our knowledge received little prior attention in the context of research on reasons people live rather than dying by suicide. Fear has been discussed in prior literature as a motivation to live, but to our knowledge it does not play a formal role in any clinical intervention strategies, although anecdotally it is used by clinicians for that purpose. For example, Linehan^71^ reports leaning into religious fear with suicidal clients, apparently even clients who did not bring religious fears to the table themselves. (“They seemed to think that their suffering would end if they killed themselves. I’d point out that there were no data proving that was true ... There are religions that believe if you kill yourself you will go to hell, and others that believe you will have to live your whole life over again. That could keep me from doing it!”) Other negative emotions like spite and anger are generally viewed as part of the problem rather than part of the solution^76–78^. Similarly, clinicians conventionally associate alcohol, illicit substances, and other potentially harmful activities with increased risk, as obstacles in the prevention of suicide.

In contrast, our analysis suggests a new look at negatively valenced emotions and activities in the context of helping individuals counter their momentum toward suicide. Without question, fear, anger, spite, alcohol, drugs, and the like are potential, and far too often real, sources of harm. But, then again, so is living in terror of a judgmental deity sending you to hell^79,80^. Our evidence suggests that things categorized clinically as obstacles to overcome may not *always* be negatives in the context of preventing suicide. The lived experiences of the respondents in this study suggest a more nuanced investigation into potential protective factors associated with negatively valenced states.

One way of making sense of this apparent dual role for negatively valenced emotions and activities—conventionally viewed as contributing to dysfunction, but also able to serve as a barrier in progress toward suicide—is to frame the question in terms of balancing immediate versus longer-term clinical treatment goals. Above we have noted the potential preventive value in short-term hedonic focus of attention. Our results suggest that even negatively valenced emotions or activities can have a similar value for some individuals, especially in crisis.

Faber and Mazlish^81^ observe, “When a person is drowning, it’s not the time to give swimming lessons”. One direction for further investigation suggested by our analysis is a deeper consideration of behaviors and modes of thought that are conventionally conceived of in terms of risk or harm, but which may serve as life preservers. In a non-crisis state, substance use, spiteful feelings, and similar thoughts and behaviors are naturally a target for careful management with the aim of reducing their prevalence and impact. It may, however, be valuable for clinicians to recognize explicitly that some of the very same factors that would otherwise weigh patients down might, in the short term, be helping to keep them afloat. One respondent wrote, “Drugs. Not gonna lie. They have almost killed me a few times but lots of times they’ve also been the reason I could stomach everything around me”. And consider the individual who wrote, “I didn’t want all those asshole childhood abusers to win”. In a non-suicidal state, a likely therapeutic goal would be to let go of the anger toward childhood abusers, to allocate that emotional energy toward things that are more productive and healthy in the long term. Here, that negative emotional energy is contributing toward motivation to not die. (We observe in particular that, unlike sadness, hopelessness, or feelings of isolation, some negative feelings involve increased energy and activation; cf. the negative valence, high arousal quadrant in the circumplex model of affect^82.^)

*The clinical importance of lived experiences.* Many clinicians lack the training they need in order to assess and intervene with people at risk for suicide^83–86^. But even among clinicians with appropriate training, an incomplete understanding of the individual’s experience and situational context can hinder the establishment of rapport and contribute to the gap between what the clinician knows to ask and what they know to listen for. The data we have analyzed, and the categories and themes that emerged, may contribute toward bridging that gap, enhancing clinicians’ ability to establish rapport by asking questions that will resonate with people in crisis. In a conversation with a patient, a trained clinician may know to look for indications of ideation, intent, plan, means; of thwarted belongness or perceived burdensomeness^4^; of evidence of frantic hopelessness^56^ or signs of rumination^56^. They may not know to pay close attention to the possibility that the patient’s pet, their video gaming, or their enjoyment of cannabis are potential positives in avoiding suicide. Especially when discussing so serious a topic as a patient taking their life, it is easy to miss or dismiss statements involving such things as irrelevant or as contributing negatively to the weighty topic at hand.

In addition, although the clinician’s emotional reaction/countertransference to a patient’s suicidal narrative is an essential diagnostic tool for determining risk, the reaction to a patient in crisis may also involve negative emotion, cause a desire to escape the patient, or trigger clinician hopelessness, potentially with negative consequences for treatment. In particular, especially for a clinician who has never been suicidal, hearing a patient in crisis say they want to live so that they can see how Game of Thrones ends, or to spite a toxic relative, can be jarring and potentially give rise to negative emotions that lead to ruptures in the therapeutic alliance^87–89^. Equipped with greater awareness of identifiable recurring categories for reasons to live among suicidal individuals, a clinician may be less likely to avoid writing off the patient as resistant, selfish, or oppositional.

*Refining the concept of perceived burdensomeness.* Our results also suggest a new way of looking at the widely discussed construct of perceived burdensomeness^4,90^. The theme of Concern for Others highlights that burdensomeness can go both ways: what kept some people from suicide was how their *dying* might put burdens, e.g. financial or emotional, on others. As such, it supports proposals to revisit perceived burdensomeness as a “transactional” construct, involving not just perceived liability (e.g. drain on financial resources) but also perceived contribution to others (e.g. financial contributions to one’s family, emotional support), as distinct concepts^91,92^.

This way of looking at perceived burdensomeness has clinical implications, as Hill et al.^92^ observe: “Evidence that perceived liability and perceived contribution operate independently as elements of perceived burdensomeness provides opportunities for preventive interventions to either *reduce perceived liability* or *increase perceived contribution*” (our emphasis). Our results take this a step further by suggesting that converse opportunities also exist: the Concern for Others theme indicates that momentum toward suicide is reduced by the perception that one’s dying by suicide would *increase liability* toward others or *reduce contributions* that promote their well-being. Broadly speaking, then, a clinician could be probing not only “Do you feel that others would be better off if you were dead?” but also “Are there ways in which others would be worse off if you were dead?”. We do not suggest that either assessment or intervention is as simple or mechanistic as determining where an individual falls on a burden/contribution scale; however, we believe that the results of Hill et al.^92^ and our own highlight the value of paying greater attention to reasons for living as a path away from action, a valuable counterpoint to the present focus on the path toward action.

### Quantitative results: reliability assessment and prevalence

Our primary goal in this study was qualitative: increasing our understanding of reasons people live, rather than dying by suicide, through an up to date examination of actual lived experiences. However, two analyses are important to include that are quantitative in nature. The first is an assessment of inter-coder reliability, since unacceptably low human agreement would raise questions about the coherence and applicability of the categories and themes. The second is an assessment of sample prevalence, since, although it cannot license conclusions about the population, such an assessment can help to support hypothesis generation and suggest directions for further investigation.

#### Reliability assessment

Because the TOPCAT process involves curation of computationally induced categories, it does not include the standard qualitative analysis process involving bottom-up identification of codes, coding a sample, assessing inter-coder reliability, discussing inter-coder disagreements, refining codes and guidelines, and then returning to code another sample, iterating until satisfying reliability is reached^see 93^, Fig. 3]. In addition, notwithstanding how widespread that conventional process is in fully manual content analysis, the coders engaged in a category development process are not independent, potentially biasing reliability estimates upward. And as Yarkoni and Westfall^94^ note, adopting out-of-sample evaluation—the gold standard in data-driven computational text research—mitigates the ever-present risk of overestimating performance in retrospective analysis, and it is standard practice in the validation of automated content analysis methods.

**Fig. 7:**
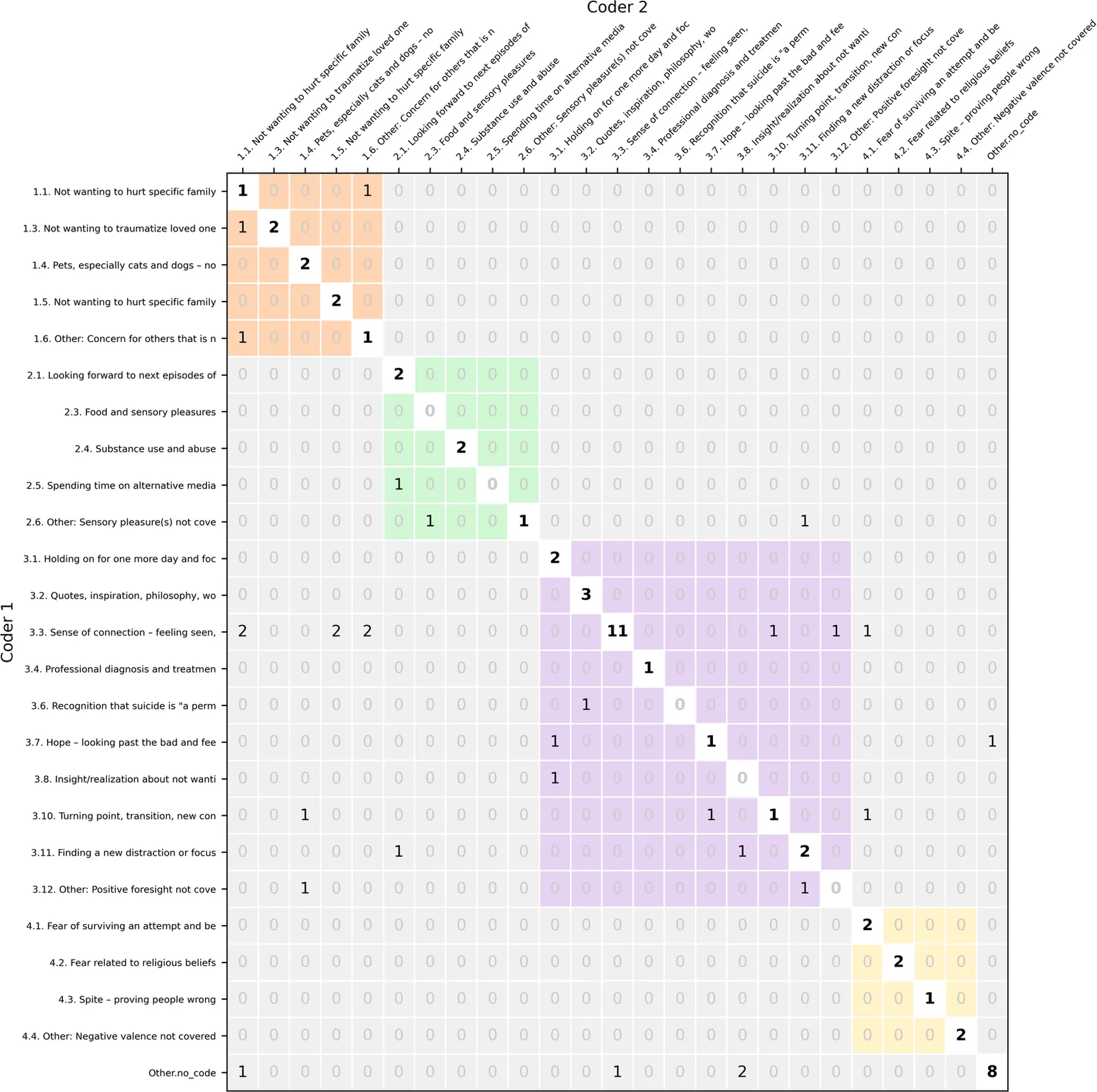
Confusion matrix for category classification under the in-common agreement criterion. The four shaded squares along the diagonal delineate sets of categories belonging to the same theme. The label Other.no_code signifies a coder choosing *Other* for theme.

For these reasons, we assessed reliability via two paid coders who were not otherwise involved in the project, on a set of 79 responses from firegate2233’s original Reddit thread that arrived after our original collection of data. (The first coder has a master’s degree in clinical psychology including research using text coding; she has received suicide prevention and emotional support training and has three years of experience providing mental health and crisis intervention support. The second coder is an expert qualitative researcher with experience on two separate suicide crisis lines and additional training in mental health.) Note that although the evaluation set is therefore “out of sample” in the statistical sense, it can be considered *representative* because it is taken from exactly the same source as the dataset from which themes and categories were induced, just for responses that came in after the original dataset’s cutoff date.

Particularly given the richness of the text responses in our dataset (recall that mean response length is a paragraph-like 62 words), it is common for multiple categories to be applicable in any given response. For each post, therefore, the coders provided a primary theme and a primary code, and optionally a secondary theme and/or code. (An “Other” theme was also added to the code set, and within each of the four themes, an “Other” code was added, e.g. *Other: Concern for others not covered by the other codes in this theme*.)

As a result there are not one but two kinds of choices per item: which code(s) to assign, and, in the case of multiple codes, which to consider primary versus secondary. Because our primary interest is in *what* categories were applicable to text content rather than their relative importance, for our main measurement of chance-corrected inter-coder agreement coders were considered to agree if any code was assigned in-common, e.g. if Coder 1 assigned primary and secondary codes A and B, and Coder 2 assigned B and C, this would be considered an agreement (based on B being coded in common). On that criterion Cohen’s *κ = 0.77* (95% CI: 0.65 to 0.88) for the five themes (including Other) and *κ = 0.59* (95% CI: 0.47 to 0.70) across 26 categories (including the other categories); see Fig. 7 for the confusion matrix. When reliability is measured by the very conservative criterion of primary-only agreement—paying attention only to the primary themes or categories and ignoring secondary (e.g. in the above example, the coders would be considered to completely disagree, despite having identified B in common)—*κ = 0.63* (95% CI: 0.49 to 0.76) for themes and 0.47 (95% CI: 0.35 to 0.59) for the 26 categories. As expected given the richness of the texts, agreement is higher under the any-match criterion, reflecting the fact that coders frequently identified overlapping but not identical code sets.

Albeit only a general rule of thumb, the *κ* thresholds for “moderate”, “substantial”, and “almost perfect” agreement are conventionally taken to be 0.4, 0.6, and 0.8, respectively^95^. Even under the (arguably too-)conservative agreement criterion, these chance-corrected agreement figures provide a reasonable degree of confidence that the categories and themes that surfaced in the qualitative analysis are coherent and make sense when applied to our sample of individuals’ experiences.

#### Sample prevalence

It must be emphasized that as a non-probability convenience sample, our dataset cannot support population-level estimates. Nonetheless, prevalence in our data may be useful in generating hypotheses and in helping to guide directions for further investigation.

The topic-modeling approach we took here, Latent Dirichlet allocation^36^, provides a straightforward way to estimate topic prevalence based on the entire text corpus being modeled. Mathematically, LDA represents each document *d* as a distribution $\theta _d$ over *K* topics, where $\theta _{d,k} = P(\text {topic } k \mid d)$ and $\sum _{k=1}^{K} \theta _{d,k} = 1$. The corpus-level prevalence of topic *k* is then estimated as the mean of the per-document topic probabilities,

1 $$\begin{aligned} \hat{p}_k = \frac{1}{N} \sum _{d=1}^{N} \theta _{d,k}, \end{aligned}$$

where *N* is the number of documents in the corpus. In our setting, *K=30* topics were fit by the original LDA model, of which we retained the 23 topics judged interpretable and on-topic by subject-matter experts in the TOPCAT curation process; the remaining seven topics are discarded. We compute $\hat{p}_k$ over the retained topics with each document’s $\theta _d$ renormalized to sum to 1 across those 23 topics

Table 1 shows prevalence at the theme level estimated from the full dataset, together with the average theme prevalences based on the human coding of 79 responses as described above. (The coder-based prevalence estimate is an average of the prevalences for the two coders.) It is important to note that the topic model provides no Other category. Therefore its estimate for Other is *0%* and probability that would be associated with that category is distributed across the four themes—and not necessarily evenly, since genuine “Other” responses might contain language aligning with any given theme to a greater or lesser extent. In addition, since the pre-curation LDA model does not disaggregate the topic of religious hopes and/or fears into religious hopes (Positive Foresight) and religious fears (Negative Valence), the corpus prevalence for that topic ($\approx 1\%$) is split evenly between those two themes. Notwithstanding those caveats, we see that the model-based estimates of prevalence in the full 16,648-document dataset are broadly consistent with human coding of the small, non-overlapping dataset taken from the same source.

**Table 1:** Theme-level prevalence estimates: human coding on the reliability dataset (*n=79*, averaged across two coders’ primary themes) and LDA model-based estimates on the full 16,648-document dataset.

Theme	Coder-based estimate (%)	Model-based estimate (%)
Concern for others	19.6	28.0
Sensory pleasures	10.1	10.1
Positive foresight	46.2	53.6
Negative valence	10.8	8.3
Other	13.3	—

LDA-based category-level prevalence estimates in the 16,648-document set are shown in Table 2, though it is important to note several caveats. First, the human-coded dataset is too small to provide validation or even a reasonable comparison point for the LDA-based prevalence estimates at this level of granularity—some codes do not appear at all in the 79-document set. In addition, as at the theme level, this prevalence estimation renormalizes over the 23 retained categories and is structurally unable to assign mass to Other; the held-out human coding suggests roughly 10–15% of responses fall outside the codebook’s specific categories, and that mass is redistributed across the 23 retained categories in the estimates shown. The within-theme Other catch-all codes likewise have no corresponding topic in the LDA model and are not represented in the table. More fundamentally, the alignment between topics and the final codes produced by the TOPCAT protocol is not exact: a topic’s probability mass for a given document reflects the topic model’s representation of that document, not a human coder’s judgment about which code or codes apply. The estimates in Table 2 should therefore be read as a first-pass, descriptive picture of category-level prevalence, useful for identifying which categories dominate the corpus but not for drawing precise quantitative conclusions about any individual category. Refining category-level prevalence estimation would require additional human coding combined with methods that adjust for classifier or coder error (e.g.^96^), and is left to future work.

**Table 2:** Category-level prevalence estimates from LDA modeling using the 16,648-document corpus.

Category	Description	Model-based estimate (%)
*Theme 1: Concern for others*		
1.3	Not wanting to traumatize loved ones, especially finding and having to deal with the body	11.0
1.4	Pets, especially cats and dogs – not wanting to hurt them, worry about them, etc.	7.2
1.1	Not wanting to hurt specific family members	5.2
1.5	Not wanting to hurt specific family members, particularly children	3.4
1.2	Financial burdens for survivors or if attempt fails	1.3
*Theme 2: Sensory pleasures*		
2.1	Looking forward to next episodes of TV, new games, experiencing new media	3.8
2.4	Substance use and abuse	2.0
2.2	Music – wanting to continue hearing it, experiencing music as meaningful, specific artists	2.0
2.3	Food and sensory pleasures	1.3
2.5	Spending time on alternative media	1.0
*Theme 3: Positive foresight*		
3.7	Hope – looking past the bad and feeling like life can get better	12.6
3.10	Turning point, transition, new connection	12.4
3.11	Finding a new distraction or focus	8.4
3.3	Sense of connection – feeling seen, heard, understood, cared about by other people	6.8
3.8	Insight/realization about not wanting to die or finding meaning in surviving an attempt	5.6
3.4	Professional diagnosis and treatment	2.6
3.6	Recognition that suicide is “a permanent solution to a temporary problem” and life will change	1.6
3.9	Insights from reading or writing, including writing a suicide note	1.2
3.1	Holding on for one more day and focusing on the next thing, routine, sleep on it and see if/how things get better in the morning	1.1
3.2	Quotes, inspiration, philosophy, wonder – things that resonate/connect	0.8
*Theme 4: Negative valence*		
4.1	Fear of surviving an attempt and being worse off	5.5
4.3	Spite – proving people wrong	2.3
*Codes not separable by the topic model*		
3.5/4.2^*^	Hope and connection with spiritual or religious beliefs / Fear related to religious beliefs	1.0

## Discussion

Our large scale analysis of reasons people did not die by suicide produces results that are overall consistent with prior work on reasons to live^16,18,23,24,97^, as well as with smaller scale analysis of the same kind of naturalistic data^37^. We interpret this alignment as evidence for convergent validity.

At the same time, there are several reasons for new understandings to emerge from the present analysis. The first is the size of the sample, which supplies more statistical evidence for patterns that might be present but hard to detect in smaller datasets. Second, this dataset is decades more recent than the most influential prior work, and in the context of evolving social norms and/or shifts in beliefs or attitudes, some of the facts on the ground about reasons to live may also have evolved. Third, reduced stigma in talking about mental health issues, along with the anonymous, general-population elicitation, may have helped avoid the problem of individuals self-censoring reasons to live that could be viewed negatively, or not responding at all. And finally, these data provide a window into respondents’ actual lived experiences—why they *did* not die by suicide, going beyond reasons why one *would* not make an attempt.

A key step toward achieving such new understandings is an ability to investigate and understand a very wide breadth of people’s lived experiences with suicidality, while at the same time recognizing that every experience is an N of 1 and progress requires aggregated analysis in order to capture generalizations. With that as the goal, language collected at a large scale is both a blessing and a curse: blessing because scale can help to uncover patterns within all the variability in experience and language, and curse because traditional content analyses at that scale are often simply too impractical to accomplish. In this work we have used computer-assisted methods to navigate that challenge, with rigorous human curation to support trust in the results.

From a theoretical perspective, our results are obtained in the context of prior work where the majority of the constructs discussed are considerations that push people toward ideation, attempts, and death in an “ideation to action” framework—widely discussed constructs of that kind include perceived burdensomeness and thwarted belonging^4^, pain and hopelessness^52^, and entrapment^56^. At the same time, as we have noted, some prior work also explicitly frames constructs identified in people’s reasons to live as being countervailing forces to such factors, e.g.^5,23,31^. For example, in discussing their three-step theory of suicide, Klonsky et al.^5^ articulate a version of such an opposition: “Whereas pain provides the push away from life *…* connectedness provides the pull toward life”. As a useful theory-independent metaphor, Bryan^31^ p. 191] refers to reasons for living as part of an “internal braking system” that opposes such pushes, and can reduce the urge to act. This more explicitly bi-directional framing has also been articulated in terms of the internal debate of suicide^30^.

Our analysis contributes to that broader, bi-directional theoretical framing by surfacing relevant constructs in a a large and recent aggregated set of suicidal individuals’ lived experiences. In some cases, we find supporting evidence for direct “pull” counterparts to established “push” concepts; for example, reasons to live within the Concern for Others theme can be seen as a counterpart to perceived burdensomeness in the interpersonal theory of suicide. In other cases, our evidence supports refinements to the theoretical discussion; for example, our analysis suggests there may be value in explicitly distinguishing interpersonal connectedness from a sense of connection to life, in contrast to the broad-umbrella reading of connectedness as a construct subsuming both in the 3-step theory of suicide. In still other cases, we identify constructs that argue for greater cross-literature theoretical engagement, e.g. connecting discussions of hedonic pleasure and eudaimonia in the psychological well-being literature to discussions in the suicidology literature of anhedonia and a possible construct we have labeled as aneudaimonia. Finally, we find empirical evidence supporting greater attention to considerations that have previously been primarily anecdotal and largely absent from the scholarly literature on suicide, such as the potential value of spite as a motivation to stay alive and the significant role of pets in people’s lives.

While the above discussion pertains primarily to the distinction between the columns in our four-quadrant description of the suicide research landscape, between reasons to die and reasons to live (Fig. 1), our results also speak to the distinction between the rows. One way to conceptualize this distinction is in terms of Bryan’s^31^ argument for a broad, high level rethinking of suicide. Bryan’s core thesis is that suicide prevention efforts are failing because they focus too heavily on framing suicide as a mental health issue. His cusp catastrophe model articulates, in addition to smooth progressions from ideation to action, the possibility of “tipping point” transitions to high-risk behavior that do not progress through the wish to die, ideation, and planning. Within his model, a reason to live—a moment of eudaimonic meaningfulness, recognizing the harm to a loved one, even a frowned-upon hedonic experience or a decision to spite someone by living—could accomplish the reverse, in the moment. We observe that this is aligned with research highlighting the value of frequent short-term assessment^98–100^, and our analysis is also aligned with the directions being pursued in newer assessments that focus on acute suicidality^10,11^ as complementary to risk over the longer term.

Clinically, our results suggest the utility of considerations that may be unanticipated or discouraged in conventional practice. Activities and mental states that are usually viewed as irrelevant, unproductive, or harmful for a suicidal person—e.g. video gaming, spite, substance abuse—apparently can serve in a productive role as short-term brakes on action. And in clinical practice, not just theory, the results also provide evidence in support of anecdotal observations that may not be systematically included in the prevention literature, such as the protective value of someone’s love for a pet, the idea that anger can provide powerful motivation to stay alive, or the idea that wanting to see the end of a miniseries can make the difference between surviving one day more or not. Clinicians already know that it is important to pay attention to a patient’s situational context, but these potential lifelines can easily be missed, dismissed as irrelevant, or even give rise to negative emotions on the part of the clinician that interfere with rapport and successful intervention.

Also from the clinical perspective, the categories we have identified may be a way to stimulate innovative thinking for interventions. Empirical support for the importance of pets to many people, for example, suggests an exploration of low-cost interventions in the spirit of “caring contacts” that have been demonstrated to reduce suicidal behaviors^101–103^. One might conjecture that for the subset of individuals with a beloved pet, simply placing the pet at the foreground of attention—e.g., looking at a picture of the pet as the last thing before going to sleep at night, and the first thing in the morning—might serve as a protective measure. More generally, our analysis of reasons to live may be valuable to clinicians as potential directions for channeling cathexis, using what we have discovered in a large body of lived experiences to help constructively direct the emotional energy of individual patients.

## Limitations

Notwithstanding the potential value of these take-aways, a number of limitations point toward the importance of interpreting our study as a starting point for future work rather than an endpoint. First, as we have noted, the Reddit-based sample here was obtained opportunistically rather than systematically, with no information about individuals other than their responses and an obvious survivor bias. Related, we are relying on self-report, and our analysis of first-person accounts assumes a degree of self-awareness and comprehensiveness in responses that is likely to vary across respondents, including cultural variation not captured within Reddit’s primarily U.S. and English-speaking user base. We also lack upstream information about how close to suicide an individual was (e.g. whether they had already made a plan or obtained lethal means) and downstream information about outcomes; for example, it is possible that contributors to this thread have made an attempt since responding or died by suicide. While these limitations are typical for mental health studies involving social media^104^, it is important that future work involve proper sampling with attention to these considerations as well as standard considerations such as demographic characteristics and diagnostic history.

Second, although our reliability assessment yielded reasonable inter-coder agreement, that assessment was done on a small 79-item sample and more coder guideline development may be warranted. For example, short responses like “my brother and grandmother” or “my little sister” could legitimately be interpreted as either *sense of connection with* with those people or *concern for* those people, and clearer guidelines could help resolve that ambiguity consistently. In addition, there are some cases where relevant categories or subcategories may not have emerged given the topic model’s selected level of granularity; for example, on inspection we find that concern for friends, not just family members, may have helped some individuals avoid making a suicide attempt. The fact that only 10-15% of the human-reviewed items were coded as Other is promising, but it is also a logical starting point for further analysis.

Third, LDA topic modeling is only one of many ways to use computer-driven inductive analysis in TOPCAT’s curated process. We selected it because it is very well established and well understood as a computational content analysis method^105,106^, in contrast to an emerging but still immature artificial intelligence (AI) literature on content analysis using large language models, where systems are black boxes and current methods leave much to be desired in terms of trustworthiness, validation, and established norms^107,108^. However, as AI progresses, revisiting this choice may be warranted.

## Conclusions

A spontaneous question on Reddit about people’s experiences surviving suicidality elicited a large set of rich, substantive responses about how people got through their dark times. Using a computer-assisted process to permit rigorous qualitative content analysis at scale, we derived empirically grounded categories and themes, used human coding to establish confidence in their sensibility and coherence via inter-coder reliability, and computed preliminary estimates of theme- and category-level prevalence in our dataset.

The results we obtained offer a number of starting points for further discussion. Our data-driven analysis yielded a reassuring degree of alignment with a number of concepts that appear prominently theories of suicide, but they also support proposed theoretical framings of suicidality not as a unidirectional progression from ideation to action but as an internal debate influenced by factors that push toward dying or pull toward living. Related, we find promising correspondences between constructs—for example, hedonic pleasures and anhedonia—that suggest value in a greater degree of cross-literature discussion among researchers who focus on suicide and researchers who focus on well-being. And, our study provides a window on people’s reasons to live that is more up to date, more experience-driven, and (thanks to Reddit’s anonymity) arguably more grounded in natural, unconstrained responses than were the field’s foundational resources dating back to the 1980s and 1990s.

As a final observation, individual-level experiences have long been a part of the study of suicide and people’s not dying by suicide. Viktor Frankl, psychologist and concentration camp survivor, observed suicidal men in the camp who were persuaded to go on because, even if they did not expect more from life, “life was still expecting something from them; something in the future was expected from them”—in one case it was the man’s child; the other was a scientist whose books had yet to be finished (echoed by a respondent in our dataset, who wrote, “I remember that if I vanish my books will be left incomplete”). Frankl’s development of logotherapy, which centers on finding meaning (cf. eudaimonia), was strongly informed by his concentration camp observations^109^.

Similarly, Marsha Linehan describes in her memoir how the contemplation of her own experiences, and the experiences of others, drove her sense of purpose and her development of dialectical behavioral therapy (DBT) as a practical therapeutic approach for suicidal people. The present study has also been inspired by the idea that, in order to prevent suicide, it is essential to understand the experiences of people who could have died by suicide, but did not. Analyzing a natural set of such experiences, orders of magnitude larger than in previous studies, is a step toward work that further elicits people’s experiences in large quantity, identifies interpretable insights, and translates those insights into advances that can help save lives.

## Data Availability

Our data will be made available to other researchers upon request subject to any requirements or constraints as determined by the University of Maryland Institutional Review Board. If permitted by the IRB, data will be made publicly available on a data sharing platform (e.g. Harvard Dataverse, Zenodo, or PsychArchives). Provision of supporting data to editors/reviewers will be made a priority upon request.
